# Supplementary material for: On the role of nucleotides and lipids in the polymerization of the actin homolog MreB from a Gram-positive bacterium
Source: eLife. 2023 Oct 11;12:e84505. doi: 10.7554/eLife.84505 (PMC10718530; doi:10.7554/eLife.84505)
Supplement: MDAR checklist [file elife-84505-mdarchecklist1.docx]

**Materials Design Analysis Reporting (MDAR)**

**Checklist for Authors**

The [MDAR framework](https://osf.io/xfpn4/) establishes a minimum set of requirements in transparent reporting mainly applicable to studies in the life sciences.

*eLife* asks authors to **provide detailed information within their article** to facilitate the interpretation and replication of their work. Authors can also upload supporting materials to comply with relevant reporting guidelines for health-related research (see [EQUATOR Network](http://www.equator-network.org/%20)), life science research (see the [BioSharing Information Resource](http://biosharing.org/)), or animal research (see the [ARRIVE Guidelines](http://www.plosbiology.org/article/info:doi/10.1371/journal.pbio.1000412) and the [STRANGE Framework](https://doi.org/10.1038/d41586-020-01751-5); for details, see *eLife*’s [Journal Policies](https://reviewer.elifesciences.org/author-guide/journal-policies)). Where applicable, authors should refer to any relevant reporting standards materials in this form.

For all that apply, please note **where in the article** the information is provided. Please note that we also collect information about data availability and ethics in the submission form.

**Materials:**

| **Newly created materials** | **Indicate where provided: section/figure legend** | **N/A** |
| --- | --- | --- |
| n/a | n/a | 🗹 |
|  |  |  |
| **Antibodies** | **Indicate where provided: section/figure legend** | **N/A** |
| n/a | n/a | 🗹 |
|  |  |  |
| **DNA and RNA sequences** | **Indicate where provided: section/figure legend** | **N/A** |
| primers. | **Listed in Table S5** |  |
|  |  |  |
| **Cell materials** | **Indicate where provided: section/figure legend** | **N/A** |
| Cell lines: n/a. | n/a | 🗹 |
| Primary cultures: n/a. | n/a | 🗹 |
|  |  |  |
| **Experimental animals** | **Indicate where provided: section/figure legend** | **N/A** |
| n/a. | n/a | 🗹 |
| n/a | n/a | 🗹 |
|  |  |  |
| **Plants and microbes** | **Indicate where provided: section/figure legend** | **N/A** |
| Plants: |  | 🗹 |
| Microbes: provide species and strain, unique accession number if available, and source. | **Listed in Table S4** |  |
|  |  |  |
| **Human research participants** | **Indicate where provided:** | **N/A** |
|  |  | 🗹 |

**Design:**

| **Study protocol** | **Indicate where provided: section/figure legend** | **N/A** |
| --- | --- | --- |
|  |  | 🗹 |
|  |  |  |
| **Laboratory protocol** | **Indicate where provided: section/figure legend** | **N/A** |
| **No other step-by-step details available in addition to the Material section** | **Describe in “Materials and methods” sections, P19-25** |  |
|  |  |  |
| **Experimental study design (statistics details) *** | | |
| **For in vivo studies: State whether and how the following have been done** | **Indicate where provided: section/figure legend.** | **N/A** |
| Sample size determination |  | 🗹 |
| Randomisation |  | 🗹 |
| Blinding |  | 🗹 |
| Inclusion/exclusion criteria |  | 🗹 |
|  |  |  |
| **Sample definition and in-laboratory replication** | **Indicate where provided: section/figure legend** | **N/A** |
| State number of times the experiment was replicated in the laboratory. | **Number of replicates and methods are indicated in the corresponding figure legends:** Figure 2A, 2B**,** 3A, 3B, 3C**,** 4A, 4B, 5A, 5B  **and supplementary figures:** 2S2A, 2S2B**,** 3S1A, 3S1C, 3S1B, 3S1D**, 3**S2B**,** 3S3C, 3S3D, 3S3E, 3S3F, 3S3G**,** 4S4A, 4S4B, 4S4D**,** 5S1A, 5S1B, 5S1C, 5S1D, 5S1E. |  |
| Define whether data describe technical or biological replicates. **All replicates throughout the manuscript are independent (not technical replicates).** |  |  |
|  |  |  |
| **Ethics** | **Indicate where provided: section/submission form** | **N/A** |
| Studies involving human participants. |  | 🗹 |
| Studies involving experimental animals. |  | 🗹 |
| Studies involving specimen and field samples. |  | 🗹 |
|  |  |  |
| **Dual Use Research of Concern (DURC)** | **Indicate where provided: section/submission form** | **N/A** |
| If study is subject to dual use research of concern regulations, state the authority granting approval and reference number for the regulatory approval. |  | 🗹 |

**Analysis:**

| **Attrition** | **Indicate where provided: section/figure legend** | **N/A** |
| --- | --- | --- |
| Describe whether exclusion criteria were pre-established. Report if sample or data points were omitted from analysis. If yes, report if this was due to attrition or intentional exclusion and provide justification. |  | 🗹 |
|  |  |  |
| **Statistics** | **Indicate where provided: section/figure legend** | **N/A** |
| Describe statistical tests used and justify choice of tests.  **A nested T-test was used to compare the distribution of length of large samples (>800) of filaments in 2 condition, over 2 replicates. The T-test test if the difference of distribution is greater between conditions than between replicates.** | **Legend of Fig3-Sup1A**. |  |
|  |  |  |
| **Data availability** | **Indicate where provided: section/submission form** | **N/A** |
| For newly created and reused datasets, the manuscript includes a data availability statement that provides details for access (or notes restrictions on access). | **On front page of submission pdf** |  |
| When newly created datasets are publicly available, provide accession number in repository OR DOI and licensing details where available.  **Structure dataset are deposited in the PDB database as 7ZPT and 8AZG** | **Result section (pages 6 and 15) and Table S1** |  |
| If reused data is publicly available provide accession number in repository OR DOI, OR URL, OR citation. |  | 🗹 |
|  |  |  |
| **Code availability** | **Indicate where provided: section/figure legend** | **N/A** |
| For any computer code/software/mathematical algorithms essential for replicating the main findings of the study, whether newly generated or re-used, the manuscript includes a data availability statement that provides details for access or notes restrictions. |  | 🗹 |
| Where newly generated code is publicly available, provide accession number in repository, OR DOI OR URL and licensing details where available. State any restrictions on code availability or accessibility. |  | 🗹 |
| If reused code is publicly available provide accession number in repository OR DOI OR URL, OR citation. |  | 🗹 |

**Reporting:**

The MDAR framework recommends adoption of discipline-specific guidelines, established and endorsed through community initiatives.

| **Adherence to community standards** | **Indicate where provided: section/figure legend** | **N/A** |
| --- | --- | --- |
| State if relevant guidelines (e.g., ICMJE, MIBBI, ARRIVE, STRANGE) have been followed, and whether a checklist (e.g., CONSORT, PRISMA, ARRIVE) is provided with the manuscript. |  | 🗹 |

* We provide the following guidance regarding transparent reporting and statistics; we also refer authors to [Ten common statistical mistakes to watch out for when writing or reviewing a manuscript](https://doi.org/10.7554/eLife.48175).

**Sample-size estimation**

- You should state whether an appropriate sample size was computed when the study was being designed
- You should state the statistical method of sample size computation and any required assumptions
- If no explicit power analysis was used, you should describe how you decided what sample (replicate) size (number) to use

**Replicates**

- You should report how often each experiment was performed
- You should include a definition of biological versus technical replication
- The data obtained should be provided and sufficient information should be provided to indicate the number of independent biological and/or technical replicates
- If you encountered any outliers, you should describe how these were handled
- Criteria for exclusion/inclusion of data should be clearly stated
- High-throughput sequence data should be uploaded before submission, with a private link for reviewers provided (these are available from both GEO and ArrayExpress)

**Statistical reporting**

- Statistical analysis methods should be described and justified
- Raw data should be presented in figures whenever informative to do so (typically when N per group is less than 10)
- For each experiment, you should identify the statistical tests used, exact values of N, definitions of center, methods of multiple test correction, and dispersion and precision measures (e.g., mean, median, SD, SEM, confidence intervals; and, for the major substantive results, a measure of effect size (e.g., Pearson's r, Cohen's d)
- Report exact p-values wherever possible alongside the summary statistics and 95% confidence intervals. These should be reported for all key questions and not only when the p-value is less than 0.05.

**Group allocation**

- Indicate how samples were allocated into experimental groups (in the case of clinical studies, please specify allocation to treatment method); if randomization was used, please also state if restricted randomization was applied
- Indicate if masking was used during group allocation, data collection and/or data analysis
